# Barriers and enablers of vigorous intermittent lifestyle physical activity (VILPA) in physically inactive adults: a focus group study

**DOI:** 10.1186/s12966-023-01480-8

**Published:** 2023-07-04

**Authors:** C. Thøgersen-Ntoumani, M. Kritz, A. Grunseit, J. Chau, M. Ahmadi, A. Holtermann, A. Koster, C. Tudor-Locke, N. Johnson, C. Sherrington, S. Paudel, C. Maher, E. Stamatakis

**Affiliations:** 1 Danish Center for Motivation and Behavior Science (DRIVEN), Department of Sports Science and Clinical Biomechanics, University of Southern Denmark, Odense, Denmark; 2 Curtin School of Population Health, Curtin University, Perth, Australia; 3 School of Public Health, University of Technology Sydney, Sydney, Australia; 4 Department of Health Sciences, Macquarie University, Sydney, Australia; 5 Mackenzie Wearables Research Hub, Charles Perkins Centre, University of Sydney, Sydney, Australia; 6 School of Health Sciences, Faculty of Medicine and Health, University of Sydney, Sydney, Australia; 7 National Research Centre for the Working Environment, Copenhagen, Denmark; 8 School for Public Health and Primary Care, Maastricht University, Maastricht, The Netherlands; 9 College of Health and Human Services, University of North Carolina Charlotte, Charlotte, USA; 10 Faculty of Medicine and Health, University of Sydney, Sydney, Australia; 11 School of Public Health, University of Sydney, Sydney, Australia; 12 School of Exercise and Nutrition Sciences, Deakin University, Melbourne, Australia; 13 Allied Health and Human Performance, University of South Australia, Adelaide, Australia

**Keywords:** Physical activity, Aging, Physical activity intensity, Exercise, COM-B model, Thematic analysis, Behavior change

## Abstract

**Background:**

Vigorous Intermittent Lifestyle Physical Activity (VILPA) refers to brief bouts of vigorous intensity physical activity performed as part of daily living. VILPA has been proposed as a novel concept to expand physical activity options among the least active. As a nascent area of research, factors which impede or encourage VILPA in physically inactive adults are yet to be explored. Such information is pertinent in the design of future interventions. We examined the barriers and enablers of VILPA among physically inactive adults using the Capability, Opportunity, Motivation, Behavior (COM-B) model as a conceptual framework.

**Methods:**

We recruited a sample of self-identified physically inactive middle-aged and older adults (*N* = 78) based in Australia to take part in 19 online focus groups across three age groups: young-middle (age 35–44), middle (age 45–59) and old (age 60–76). We analyzed interviews using a critical realist approach to thematic analysis. Identified barriers and enablers were subsequently mapped onto the COM-B model components.

**Results:**

The data generated 6 barriers and 10 enablers of VILPA that corresponded to COM-B concepts. Barriers included physical limitations (physical capability), perceptions of aging, need for knowledge (psychological capability), environmental constraints (physical opportunity), perceptions of effort and energy, and fear (automatic motivation). Enablers included convenience, reframing physical activity as purposeful movement, use of prompts and reminders (physical opportunity), normalization of taking the active option, gamification (social opportunity), sense of achievement, health improvements, personally salient rewards (reflective motivation), identity fit, and changing from effortful deliberation to habitual action (automatic motivation).

**Conclusion:**

The barriers and enablers of VILPA span capability, opportunity, and motivation beliefs. Promoting the time-efficient nature and simplicity of VILPA requiring no equipment or special gym sessions, the use of prompts and reminders at opportune times, and habit formation strategies could capitalize on the enablers. Addressing the suitability of the small bouts, the development of specific guidelines, addressing safety concerns, and explicating the potential benefits of, and opportunities to do, VILPA could ameliorate some of the barriers identified. Future VILPA interventions may require limited age customization, speaking to the potential for such interventions to be delivered at scale.

**Supplementary Information:**

The online version contains supplementary material available at 10.1186/s12966-023-01480-8.

## Background

Physical activity is a key contributor to physical and mental health and well-being [1, 2]. However, large sections of populations across the world do not meet current physical activity guidelines [3], and levels are particularly low among middle-aged and older adults [4, 5]. Further, physical activity levels have been stagnant over the past decade [6]. A systematic review of 162 quantitative and qualitative studies involving middle-aged and older adults revealed that environmental factors and resources (e.g., cost, weather, and facilities), difficulty in regulating physical activity behavior, and lack of beliefs in capabilities were commonly reported barriers [7]. Lack of motivation, pain or physical discomfort, poor health, fatigue and lack of energy, and lack of time are also consistently reported as pertinent physical activity barriers for adults [8, 9]. Therefore, interventions which can address or ameliorate these barriers are warranted.

Physical activity guidelines recommend that adults should undertake a minimum of 150–300 min of moderate-to-vigorous physical activity (MVPA) per week [10]. Recently, the minimum bout length requirement has been removed [11], which offers new opportunities for middle-aged and older adults to increase their levels of physical activity in shorter bouts which may be more suitable for people with health problems or those who report time or capability as barriers to be physically active.

Vigorous Intermittent Lifestyle Physical Activity (VILPA) [12] refers to brief, vigorous bouts of incidental physical activities lasting 1 or 2 min that are done during activities of daily living, such as carrying shopping bags, carrying children, or walking uphill [13]. VILPA offers a more flexible approach to being physically active than traditional structured exercise and does not encroach on people’s time, nor does it require preparation or access to facilities, thus circumventing some identified barriers (e.g., time, perceived lack of resources) to being physically active [7–9] and allowing people to achieve physical activity guidelines in a time-efficient manner. Furthermore, VILPA at levels below those recommended by the guidelines appears to offer meaningful health benefits. For example, recent evidence from a sample of non-exercisers in the UK Biobank showed that three VILPA bouts per day (lasting the equivalent of one or two minutes each) were associated with a 38–40% reduction in all-cause and cancer mortality risk and a 48–49% reduction in cardiovascular disease (CVD) mortality risk [12]. As little as 4.4 VILPA minutes per day was associated with a 26–30% reduction in all-cause and cancer mortality risk and a 32–34% reduction in CVD mortality risk. Similar findings have been reported for incidence cancer [14]. These striking effect sizes support the potential of VILPA as an alternative to structured exercise and another, potentially feasible, option to accrue health benefits from physical activity. Other analogous concepts involving short bouts of physical activity of moderate-to-vigorous intensity, such as ‘exercise snacks’ [15], have been proposed. However, only VILPA places emphasis on integration into everyday activities, and is the only type of short bout vigorous physical activity that is supported by epidemiological evidence [12].

As VILPA is embedded into activities of daily living, future interventions should focus on (1) identifying new opportunities to do VILPA (e.g., parking further away and carrying shopping instead of using a trolley in the supermarket), and (2) empower and support individuals to increase the intensity of their existing daily activities (e.g., leave later from home to catch the bus to encourage fast walking). To enable effective intervention development, it is important to delineate factors that hinder or facilitate the behavior. Due to the novelty of the concept, the barriers and enablers of VILPA are yet to be explored. Therefore, the aim of this study was to identify barriers and enablers of VILPA which can help identify suitable intervention targets to use VILPA as a means of promoting health and well-being in physically inactive adults.

## Method

### Study design

As part of a larger program of research, we conducted a qualitative and video-conferencing enabled focus group interview study examining the feasibility of VILPA among middle-aged and older adults. Ethics approval was provided by Curtin University’s Human Research Ethics Committee (HRE2020-0670).

### Recruitment

Australian participants (35–76 years of age) were drawn from a world-wide survey examining the socio-demographic and health correlates of VILPA bouts (results of which are not reported here). Only participants who self-identified as physically inactive were eligible to take part in the survey and therefore the subsequent focus group interviews (see Additional File 1 for the screening questionnaire). Recruitment for the initial survey took place via social media, online newsletters, and the distribution of flyers in universities and medical practices. Other recruitment strategies included a radio broadcast and snowball sampling. Participants from the survey who were based in Australia were asked if they would be willing to take part in focus group interviews to explore their experiences and beliefs about VILPA. A total of 190 individuals initially expressed interest in participating in the focus groups. To ensure inclusivity, all 190 individuals were invited to sign up for a focus group, providing an opportunity for anyone interested to contribute their perspectives. Participants could sign up for a focus group via an online event managing website (Eventbrite), and spots were allocated on a first-come, first-served basis. To achieve diversity within the sample, and capture a range of perspectives, purposeful sampling techniques were used. Specifically, efforts were made to ensure representation across different age groups by offering a similar number of focus group spots for each age group. The final sample size was determined based on practical considerations and the desired level of data saturation. Our goal was to reach a point where additional participants would not substantially contribute new insights or themes. To ensure diversity within the sample, we aimed to recruit 25–27 participants for each age-group (i.e., a final sample of about 75–78). This was achieved by controlling the number of focus group spots offered, thereby ensuring an equitable distribution across age groups. All the participants were provided with information about the study and signed informed consent was obtained.

### Data collection

Data on socio-demographics characteristics (age, gender, state, ethnicity, highest level of education, and marital status) and health (health status, health issues, and weight status) were self-reported in the survey part of the study. Further, based on the participants’ self-reported postcode, we retrieved each participant’s Socio-Economic Indexes for Areas (SEIFA) score which is an indicator of relative socio-economic advantage or disadvantage. Scores are based on quintiles ranging from 1 (most disadvantaged) to 5 (most advantaged).

### Focus groups

Participants were organized into focus groups based on age: young (age 35–44), middle (age 45–59) and old (age 60–76). This was done as individuals feel more socially connected to people who are in some way similar to themselves [16], which could facilitate more openness in sharing their beliefs and experiences. Focus groups were chosen because they allow for reflections to be shared by participants which could then stimulate discussion among them.

Typically, focus group interviews consist of between 6 and 12 people [16], and while we had planned for this size, most groups were smaller (2–7 participants plus the interviewer/facilitator), several participants did not attend their assigned slot and interviews had to be re-scheduled. A total of 19 focus group interviews were conducted. Video-conferencing enabled focus groups were implemented due to public health restrictions on gatherings and movement imposed during the COVID-19 pandemic and because the participants were spread across Australia. With participant consent, sessions were recorded (audio and video) and the audio was transcribed by a professional service.

The COM-B (Capability, Opportunity, Motivation and Behavior), a model that forms the core of the Behavior Change Wheel [17], served as the guiding framework for focus group implementation and analysis (see below). The COM-B framework argues that behavior may be understood as the outcome of a person’s capability (physical and psychological), opportunity (physical and social) and motivation (reflective and automatic). The COM-B framework has previously been used to examine barriers and enablers of physical activity in a number of life contexts, such as in pregnant women with overweight and obesity [18], women with gestational diabetes [19], and adolescents with intellectual disabilities [20]. A copy of the interview guide mapped to the COM-B domains is provided in Additional File 2. The interview guide was developed to explore capability, opportunity and motivation beliefs about VILPA, given COM-B was the guiding framework.

A PowerPoint presentation was used to explain the concept of VILPA to the participants. The presentation included examples of VILPA, and subsequently, participants were then encouraged to generate their own examples of VILPA. This approach ensured that the researcher could verify that all participants had a comprehensive understanding of the concept. To enhance comprehension among participants, VILPA was described as “short daily movement.“ This choice of terminology aimed to employ language that was familiar and relatable. The focus group interview guide was pilot tested with one focus group and subsequently refined. The duration of the interviews ranged from 81 to 95 min.

The second author (PhD, female, research associate, three years’ experience conducting qualitative research) conducted the focus groups. No prior relationship existed between the interviewer and the participants. The first author observed three of the interviews. The second author wrote reflective notes following each interview which were used to facilitate the analysis. The participants were compensated with a $30 dollar Amazon voucher to acknowledge their time.

### Analysis

The analysis was conducted in NVivo [Version 12, QSR International, Melbourne, Australia] by the first author. We used a critical realist approach to thematic analysis [21], which combines ontological realism and interpretivist epistemology [22]. We focused on the development of experiential themes which refers to participants’ viewpoints, intentions, beliefs, and experiences as they are evident in the data.

The six-phase analytical process as outlined by Braun and Clarke [23] was followed. First, in the familiarization phase, the first author read and re-read transcripts numerous times and made initial reflections about patterns perceived in the data. In phase 2, the first author generated initial codes which included short descriptions or labels assigned to the data that were relevant to the research question. In phase 3, initial themes were constructed which represented the aggregated meanings across the whole dataset. In phase 4, the themes were reviewed to ensure they were distinct and informative in relation to the research question. This phase included the use of the ‘critical friend’ approach [24] whereby the second and third author acted as “sounding boards” and critically appraised the themes developed by the first author, followed by a discussion which meant the addition of some new themes and revision of the definitions of other themes. Phase 5 involved the first author (re)defining and naming themes, ensuring the dual criteria outlined by Patton [25] such that each theme provided a coherent and internally consistent account of the data which is distinct from other themes. Phase 6 consisted of producing the report, which in reality, was done iteratively during previous phases too.

Finally, a deductive approach was used whereby the different themes were mapped unto one of the COM-B dimensions as relevant. To ensure analytical transparency, the first author made notes of all the methodological decisions and reflected on possible biases during the interpretation phase. As per previous examples in the literature [e.g., 26], barriers and enablers were analyzed separately. The Consolidated Criteria for Reporting Quality Research (COREQ) [27] was used to ensure thorough reporting of the analytical process (see Additional File 3). We included interpretations of each theme when reporting them in the results section.

## Results

### Participants

Seventy-eight participants (19.4% of the total amount of participants from the worldwide survey) engaged in the focus group interviews. The socio-demographic and health characteristics of the participants are presented in Table 1.

**Table 1:** Summary characteristics of focus group participants

Characteristic	%	*n*
Age (years) (self-reported)		
35–44	33.33	26
45–59	32.05	25
60–76	34.62	27
Gender (self-reported)		
Female	75.64	59
Male	23.08	18
Other	1.28	1
State (self-reported)		
Western Australia	29.49	23
South Australia	8.97	7
Queensland	11.54	9
Victoria	19.23	15
Tasmania	2.56	2
New South Wales	24.36	19
Australian Capital Territory	1.28	1
Northern Territory	1.28	1
Norfolk Islands (External territory of Australia)	1.28	1
Ethnicity (self-reported)		
White	73.08	57
South-East Asian	11.54	9
Southern and Central Asian	3.85	3
North African/Middle Eastern	1.28	1
Sub-Saharan African	1.28	1
Other- not specified	8.97	7
Highest level of education (self-reported)		
Year 11 or below (incl Certificate I & II)	10.26	8
Year 12/High school diploma or equivalent	6.41	5
Diploma/Advanced Diploma/Vocational training	23.08	18
Bachelor/Masters/Graduate diploma	53.85	42
Doctoral degree/PhD	3.85	3
Other	2.56	2
Self-reported health status		
Poor	11.54	9
Fair	16.67	13
Good	39.75	31
Very good	24.36	19
Excellent	7.69	6
Self-reported health issues		
Yes	48.72	38
No	51.28	40
Self-reported weight status		
Very underweight	2.29	1
Slightly underweight	2.29	1
About the right weight	15.38	12
Slightly overweight	55.13	43
Very overweight	26.92	21
Marital status (self-reported)		
Never married	16.67	13
Married	50	39
Divorced or separated	23.08	18
Widowed	3.85	3
Other	6.41	5
SEIFA score		
1 = Most disadvantaged	5.13	4
2	20.51	16
3	20.51	16
4	14.10	11
5 = Most advantaged	25.64	20

*Note*. SEIFA = Socio-Economic Indexes for Areas

In brief, most participants were female, white, and most perceived themselves to be in good or very good health and to be overweight. Socio-economic status (SEIFA scores) ranged from low (1) to high (5).

### Main results

An overview of all the themes, codes and illustrative quotes matched to the COM-B components are presented in Table 2 (barriers) and Table 3 (enablers).

**Table 2:** Barriers: Themes and illustrative quotes mapped to COM-B

COM-B dimension	Codes	Theme	Illustrative quote	Age groups (FG)#
Capability - physical	Health problems, illness, injury, pain	Physical limitations	I have a back injury and a neck injury. That stops me from doing short, sharp movements. I tend to try and just work through the pain if I’m doing something. If I’m walking or bending and I get pain, I tend to just try and work through that pain. I've got to admit that I don’t necessarily put myself in that same position again, to avoid going through that pain again (Female, age 61, good health, very overweight*, FG13).	Young (FG3, FG10, FG19) Middle (FG6, FG7, FG9, FG16) Old (FG2, FG13, FG17)
Capability - psychological		Perceptions of aging	Given my age, I'm thinking, "Well, you know, do I really need to keep my, um, exercise up, and... and my routine, because at the end of the day, is it going to help me?" (Male, age 59, good health, slightly overweight, FG9)	Middle (FG6; FG9) Old (FG2, FG14, FG17)
	Unsure of benefits, unsure of how much to do, unsure of how, unsure of how to gauge intensity	Need for knowledge	But maybe, that’s what I need to do at the end, at the, make the, the walk up the hill at the end of every, um, every walk. Make sure that that’s what I do, just to bring that last bit in. I mean, high intensity, for how long? How long are you supposed to do it for? How often? Is it every day? Every walk? Once a week? Um- (Female, age 71, excellent health, slightly overweight, FG1)	Young (FG4, FG5, FG10) Middle (FG1, FG6, FG9, FG11, FG15, FG16) Old (FG8, FG12, FG17)
Opportunity – physical	Bad weather, hills, living location, looking for opportunities	Environmental constraints	The mall that’s within two kilometers of my house doesn’t have stairs in it. It’s all on the same level, so that takes stairs out of the equation. I live in a single level house; I don’t have stairs. It puts you in a bit of a dilemma with regards to a lot of the stuff. From what I can see, you’re asking us to do this stuff, or about how to do these things, which I’ve always done (Female, age 60, good health, slightly overweight, FG13)	Young (FG3, FG4, FG5, FG10, FG18, FG19) Middle (FG1, FG6, FG7, FG9, FG11, FG15, FG16) Old (FG2, FG8, FG12, FG13, FG14, FG17)
Motivation - automatic	Perception of low fitness or strength, body weight, dislike being out of breath	Perceptions of effort and energy	like if you’re over exhausted at work, even though my work is mostly sitting down because I work at office and you know, you had such a hard day. And you have customers etc, etc and yeah you come home. You don’t want to do anything, you don’t want to move yourself. Even though you weren’t moving as much, you know. That’s prevent me. I think the daily stress, daily stress and busyness it prevents me from ah, you just want to lay down in a bed and watch TV or something just to you know, distract yourself from the stresses. I think the stresses play a big factor as well (Female, age 44, very good health, slightly overweight, FG4).	Young (FG3, FG4, FG5, FG10, FG19) Middle (FG1, FG6, FG9, FG16) Old (FG2, FG12, FG14, FG17)
	Fear of injury, fear of falling	Fear	Yeah, we go high intensity with the fast walking. I'm always a bit worried, if I really up my pace with my walking that I’m gonna stumble and fall. I don’t know, I can’t run. I've never been able to run, never been a runner. But I can step it out, but I, if I’m gonna do the high intensity and really go fast, I'm really at risk of, I feel like I’m gonna fall (Female, age 71, excellent health, slightly overweight, FG17)	Young (FG10) Middle (FG6, FG16) Old (FG2, FG8, FG12, FG17)

*Note.* #this reflects the age group where statements were identified that matched the relevant theme

*this refers to self-perceived weight status

FG = Focus Group; Young = 35–44 years old; Middle = 45–59 years old; Old = 60–76 years old

**Table 3:** Enablers: Themes and illustrative quotes mapped to COM-B

COM-B dimension	Codes	Theme	Illustrative quote	Age groups (FG)#	
Opportunity – physical	Convenience, killing two birds with one stone, can get things done quickly, completing unpleasant things faster, time utilization	Convenience	for me it’s just convenience. Uhm the shops are just five minutes away. Easier to walk than to go down to the garage and take out the car and drive (female, age 38, good health, slightly overweight, FG4)	Young (FG4, FG5, FG18) Middle (FG1) Old (FG8, FG14, FG17)	
	Avoiding the gym, means to an end, what counts as physical activity, being mindful of opportunities, purposeful activity	Reframing physical activity	I guess, um, like I, I do carry the shopping around the shops and it hadn’t occurred to me that that was actually a short daily intense movement. But, you know, by the time you’ve got a few liters of milk and then a bag of flour and something like that, it’s quite heavy, (laughs), to carry these bags of shopping around (female, age 62, very good health status, about the right weight, FG17)	Young (FG5, FG10, FG18) Middle (FG1, FG6, FG7, FG15) Old (FG2, FG8, FG12, FG13, FG14)	
	Remind oneself to pick up the pace, reminders are useful, being mindful of opportunities	Use of prompts and reminders	I think those incremental reminders through your watch or your Fitbit or whatever, for me, are really handy. The little reminders of go for a three minute or two-minute walk and you’re reaching your goal for the day, you’re on a good track to reach your goal for the day, those little reminders to go "Okay, only two minutes. Right. Okay. I will go and do this now." It doesn’t have to be going, if it’s 20-minute walk, it might be a different thing. Just those little reminders throughout the day that vibrate on your wrist are a great motivator for me (Female, age 53, very good health, very overweight, FG11)	Young (FG10, FG18) Middle (FG1, FG11) Old (FG2, FG12, FG13)	
Opportunity – social	Doesn’t want to be the odd one out, norms	Normalization of taking the active option	When you’re on your own, your decision-making, uh, may be different, or you may be less inclined to move more, but if you’re in a group situation and ... You know, for example, everyone is taking the stairs, of course, you don’t want to be the odd, odd one out. And, um, that social aspect remains where you can chat to each other as you climb the stairs. And it’s integrated into sort of your life. So, if you’re out on a day with your girlfriends at the shopping center. So, it has more incentive in a group situation to follow the herd, I think. (Female, age 41, very good health, slightly overweight, FG5)	Young (FG3, FG4, FG5) Middle (FG1, FG6, FG7, FG11, FG16) Old (FG2, FG8, FG13, FG14, FG17)	
		Gamification	when you’re counting steps after uhm, I would ah take steps instead of lift because those also count towards your steps. Uhm, so yeah I would definitely do that if you were like counting steps for say a competition with your friends or with your family. I would do that. (Female, age 35, good health, slightly overweight, FG4)	Young (FG4, FG5) Middle (FG1, FG6, FG11)	
Motivation – reflective	Feeling good, intensity as an indicator of achievement	Sense of achievement	I've started doing some more around that, around that, n-, here, I think, I haven’t long been, well, I, I wasn’t born in the area so I’m just exp-, experimenting the area now. And I’ve actually found some hills and I’m actually gonna walk up and down them, because the flat ground, it doesn’t give me enough. I do walk it every day, sort of, but I find the flat, well it isn’t, doesn’t give me enough, it doesn’t make me puffed out so a, a hill actually makes me puffed out. Then I feel as though I’ve done some work. (laughs) (Female, age 71, excellent health, slightly overweight, FG17)	Young (FG18) Middle (FG1, FG5, FG6, FG9, FG11, FG15, FG16) Old (FG2, FG8, FG12, FG13 FG14, FG17)	
	Benefits of VILPA, health benefits	Health improvements	I used to set small challenges for myself, you know. I used to time myself because it’s quite a big hill, but I don’t do that anymore. So, I... I think initially I used to do it, but... but I... I believe it’s good for me, so I keep on doing it, and... and I enjoy it. So if I don’t believe there’s any benefit, I'll stop it, but I do believe there’s... it’s beneficial for me, for my health (Male, age 59, good health, slightly overweight, FG9).	Young (FG18) Middle (FG1, FG5, FG9, FG11, FG16) Old (FG2, FG12, FG13, FG14)	
	Rewards, intrinsic rewards	Personally salient rewards	Can you make it five minutes? That’d probably be better for me. (laughs) I actually find that if, if I’m doing something like if I need to do house work, um, I actually break it up into, um, separate activities that I’ll do maybe try and do 20 minutes or maybe half an hour of intense something and then take a 10 or 15 minute break. And then do the same thing repeatedly. So, so I find, I find that actually makes me move a bit quicker, uh, or makes me more inclined to get things done because it’s, it’s like, “Ah, I've only got, I've only got half an hour of this to and then pause.” And then you go again with, “Oh, I've only got half an hour of this to do and then pause again.” (Male, age 45, poor health, very overweight, FG15)	Young (FG18) Middle (FG15) Old (FG8, FG12, FG14, FG17)	
Motivation - automatic	Better aligned with lifestyle, VILPA is more natural, prefers VILPA over other activities, focus away from the activity	Identity fit	I don’t like exercise. I've never been to a gym and, and I don’t want to go either. Uhm, so for me uhm again, being able to do this gym style huffing and puffing and increasing the heart rate through natural means, to me is more beneficial (Male, age 54, very good health, slightly overweight, FG1)	Young (FG19) Middle (FG1, FG6) Old (FG12, FG17)	
	Creating a habit, restructuring daily routine, not thinking about it	Changing from effortful deliberation to habitual action	I think the pennies just dropped for me is that it may not necessarily be about incorporating more of this into your daily routine. It may also be about... It, it’s about changing your daily routine to incorporate more of this activity (female, age 49, fair health, slightly overweight, FG15)	Young (FG5, FG10, FG18, FG19) Middle (FG11, FG15, FG16) Old (FG2, FG8, FG17)	

*Note.* #this reflects the age group where statements were identified that matched the relevant theme

FG = Focus Group; Young = 35–44 years old; Middle = 45–59 years old; Old = 60–76 years old

### Barriers

A total of six themes pertaining to barriers were developed. The experiences and beliefs that acted as perceived barriers to VILPA were matched to all three dimensions of the COM-B, namely capability (physical and psychological), opportunity (physical), and motivation (automatic).

**Capability**. *Physical limitations* included pain, injuries (e.g., back), and chronic health conditions which hindered intense movements. Some participants reflected on how they had to prioritize or make ‘smart choices’ about the types of activities they engaged in on a daily basis. Given the age of many of the participants, this finding is unsurprising, and is concordant with evidence on physical health barriers acting as barriers to engagement in general physical activity in middle-aged and older adults [28].

In terms of psychological capability, *perceptions of aging* were important. Some of the older participants explained their level and ability to do VILPA in terms of perceived physical limitations due to age. For example, some questioned whether VILPA was really worth the effort given their age, and others expressed a sense of disappointment that they did not think they could do VILPA. Some participants questioned if their aging bodies were suited to VILPA and believed increasing the intensity of activities ‘too much’ could be harmful. This finding aligns with a discourse of aging that highlights physical vulnerability over agency (or their ability to make their own choices and/or self-direct). Thus, it may be important to cultivate positive self-perceptions of aging, which have been shown to be associated with improved functional health, well-being and longevity [29].

Common themes explaining the perceived relative feasibility of VILPA included: a *need for knowledge* in relation to the specific benefits of VILPA, the minimal needed frequency to improve health, strategies to incorporate VILPA into their daily lives, and how to gauge whether or not they had achieved the “right” intensity. These findings depicting relative ignorance related to VILPA is likely due to the nascent nature of this concept and the absence of any relevant guidelines.

**Opportunity**. *Environmental constraints*. Many participants across the age spectrum noted how their living environment was not conducive to VILPA (for example, a lack of stairs in their house or a lack of hills in their neighborhood). Indeed, reviews have shown that the physical environment plays an important role to the promotion of physical activity, especially in older adults [30]. Other work has illustrated that the built environment plays a more important role to physical activity for individuals with low affective judgement (i.e., low enjoyment) of physical activity [31]. However, this perceived lack of opportunities in the environment also appeared to result from a lack of knowledge about, and awareness of, opportunities to do VILPA activities in any environment. For example, maximizing walking pace, lifting, and carrying things could arguably be done in most environments.

**Motivation**. *Perceptions of effort and energy* was a dominant theme for participants when explaining why they could not participate in VILPA. Participants spoke about how their perceived low level of fitness or strength or their high body weight meant that VILPA would require a high level of effort that they were not willing to invest. As VILPA was a relatively unfamiliar approach to conceptualizing physical activity for many, this may be indicative of an overprediction of discomfort [32] in relation to physical activity in general. The Theory of Effort Minimization in Physical Activity (TEMPA) [33] suggests that automatic negative affective reactions to physical activity and the natural inclination to minimize effort can override any positive intentions to be physically active.

*Fear* of injury and falling associated with high intensities, particularly in relation to walking quickly, was evident among some participants across all ages, although primarily among the older age groups. They described how abstaining from highly intense physical activity such as VILPA could avert injury and falling, and hence in essence “protect” their health. Such fears are prevalent in older adults [34] but also exists among younger adults with obesity [35]. The Fear Avoidance Model argues that individuals can catastrophize about threats to health or function (such as pain or injury), which leads to a spiral of fear and activity avoidance [36].

### Enablers

Ten themes relating to enablers of VILPA were generated, which mapped to the opportunities (physical and social) and motivation (reflective and automatic) factors of the COM-B framework (see Table 3).

**Opportunity**. *Convenience* was a prominent enabler among many of the participants. They noted how they were able to get other meaningful activities done (e.g., playing with children, cleaning the house) while doing VILPA. Indeed, the premise of VILPA is that it is vigorous physical activity incorporated into people’s existing lifestyles [13]. For example, one of the participants noted how VILPA could be done “anywhere, anytime”. Other participants described how completing what they described as “unpleasant” things could be done faster when doing VILPA.

*Reframing physical activity* as purposeful movement performed as part of everyday life was a salient theme across the different age groups. Many participants described how they disliked the idea of going to the gym and doing structured exercise which requires planning, self-regulatory effort, and time which they were not willing, or felt able, to do. Viewing physical activity as a by-product of daily life activities was helpful in motivating participants to do more VILPA. VILPA was seen as purposeful movement and thus valued as a type of physical activity. This led some participants to note that it helped them “avoid the guilt” of not exercising.

The *use of prompts and reminders* was perceived by some participants to be a potentially helpful aide to facilitate VILPA. The use of such reminders were discussed in relation to reminding them of opportunities to do VILPA, and to achieve the needed amount of VILPA to benefit their health. Indeed, previous research has shown that periodic prompts and reminders can improve the effectiveness of health behavior interventions [37].

*Normalization of taking the active option* related to the salience of social norms and not wanting to be the “odd one out”. For example, if most other people took the stairs instead of the lift, it encouraged participants to do the same so as not to be an anomaly. Social norms is incorporated as a salient determinant of behavior in various in behavioral theories (e.g., the Theory of Planned Behavior) [38], and empirical evidence also shows that social norms correlate with moderate-to-vigorous physical activity and volitional walking [39].

*Gamification*, that is incorporating elements of friendly competition, was perceived by some participants to have the potential to facilitate increases in VILPA. According to a number of participants, gamification was a way to experience a sense of achievement, and for others it served as a means of connecting with others. The role of gamification in facilitating physical activity behavior change is supported by a meta-analysis which has shown that gamified interventions overall have small-to-medium sized effects on physical activity behavior in different groups, including healthy adults and adults with chronic diseases [40].

**Motivation**. Many participants across the age spectrum noted how doing VILPA could help them feel a *sense of achievement* when they felt they were completing a challenging (evidenced by their “huffing and puffing”), yet feasible, activity. Several participants described how they “felt good” after doing VILPA and that it was a rewarding experience. Feelings of achievement align with perceptions of competence which is one of the fundamental psychological needs according to Self-Determination Theory [41]. When individuals experience satisfaction with the need for competence (as well as autonomy and relatedness), they are more likely to be autonomously motivated for the behavior and therefore maintain it in the longer-term [42].

VILPA was perceived by many participants to facilitate overall *health improvement*. For many, health improvement was the most dominant motivator. Health improvement was discussed in relation to a broad array of topics ranging from “feeling healthy” to regulating blood sugar levels and experiencing acute increases in well-being. Health improvement is an established salient motive for physical activity [9].

*Personally salient rewards* was broadly perceived to facilitate VILPA. Some participants described how the promise of a personally salient reward (e.g., having a coffee, or engaging in relaxing activities such as reading a book or watching movies) motivated the completion of VILPA activities. These rewards were of a tangible nature; that is activities that are detached from VILPA itself, as opposed to innate rewards associated with doing VILPA. Creating an expectation of rewards can incentivize behavior change and is an established behavior change technique according to the Behavior Change Wheel [17].

VILPA appeared to align well with the identity of many participants (i.e., *identity fit*), as it was more commensurate with their lifestyle and goals. Participants described their aversion to “exercise” and “the gym”, and how they felt that VILPA was a more “natural” way of being physically active. Experimental research has shown that individuals are most likely to engage in health behaviors if they are congruent with their social identities [43, 44]. Further, when behavior is congruent with identity, more positive emotions are likely to ensue [45], which in turn may facilitate continued behavioral engagement [46].

*Changing from effortful deliberation to habitual action*. Many participants commented that it would be helpful to restructure their daily routines to incorporate more VILPA, so it would become embedded as a natural part of their day. It is well established that developing regular routines in response to cue-behavior plans can be helpful in the formation of habits [e.g., 47]. The formation of habits involves developing a mental association between specific cues and behavior which is facilitated by repetition of the behavior. It involves a switch from slow (deliberate) to fast (automatic) thinking, whereby a cue automatically triggers the initiation of a behavior without the need for conscious effort, which is psychologically taxing. Engaging in the behavior thus becomes less effortful and is more likely to be sustained [48]. VILPA could be particularly well suited to the development of habits because it is embedded within activities of daily living which by definition are often habitual as they are performed repeatedly and in stable contexts.

**Table 4:** Suggestions to address barriers and enablers of VILPA in practice and research

**Barriers**	
Physical limitations	• Explore which VILPA activities can be feasibly done for individuals with different physical limitations
Perceptions of aging	• Provide education on challenging aging stereotypes • Emphasize the importance of *relative* (increasing intensity over and above what they normally do), as opposed to absolute (comparing to others or their younger selves), intensity in the promotion of VILPA
Need for knowledge	• Develop specific guidelines on VILPA and how to integrate VILPA into everyday activities
Environmental constraints	• Provide environmental cues relevant to diverse settings
Perceptions of effort and energy	• Create opportunities for individuals to experience positive affect as a result of doing VILPA
Fear	• Address injury and fall concerns • Provide VILPA safety guidelines
**Enablers**	
Convenience	• Emphasize that VILPA can be done anytime, anywhere
Reframing physical activity	• Highlight that people can experience physical activity benefits through purposeful movement
Use of prompts and reminders	• Remind individuals at the right time and place to do VILPA (e.g., when located near stairs in a shopping center)
Normalization of taking the active option	• Develop social norms (via e.g., messages and campaigns) to encourage individuals to do VILPA
Gamification	• Incorporate gamification elements into mobile applications and programs designed to increase VILPA
Sense of achievement	• Suggest ways in which individuals may feel a sense of achievement from doing VILPA
Health improvement	• Highlight the specific health outcomes of VILPA, including how much is needed to achieve those outcomes
Personally salient rewards Identity fit Changing from effortful deliberation to habitual action	• Encourage the use of personally salient rewards • Use communication strategies (e.g., on social media) that emphasize how VILPA aligns with the identity of non-exercisers • Encourage the development of routines by identifying context-specific triggers/cues that elicit specific VILPA activities

## Discussion

We identified numerous barriers and enablers of VILPA, many of which overlap with previous research on barriers and enablers of physical activity in general [7]. The barriers we identified that were related to lack of knowledge, health problems and physical limitations, fear of injury and pain, perceiving physical activity as taking too much effort, not prioritizing physical activity, environmental constraints, and perceptions of aging were similar to previous studies. However, a range of novel enablers were identified in our study which have not been identified in previous research. These include reframing physical activity, convenience, personally salient rewards, and identity fit. For example, the nature of VILPA allows for a reframing of physical activity as activities that are functional and compatible with people’s priorities and values. Our results highlight the complexity of possible determinants of VILPA behavior change.

Consistent with the COM-B approach, the results of our study can be used to inform a behavioral diagnosis, which, in turn, will be instrumental in the design of interventions to promote VILPA among physically inactive middle-aged and older adults. While some of the identified themes may be challenging to target in interventions to increase VILPA (e.g., physical limitations and environmental constraints), our results highlight numerous factors that are potentially modifiable via interventions. These include perceptions of aging, need for knowledge, perceptions of effort and energy, fear, reframing physical activity, use of prompts and reminders, normalizing VILPA, adding gamification elements, and changing from effortful deliberation to habitual action.

In interpreting our results, it is important to consider that most participants perceived (> 80%) themselves to be overweight. Reframing physical activity as purposeful movement and avoiding the guilt of not “going to the gym” may be more important for people who consider themselves overweight. This is because weight stigma can be prevalent in gym settings and may therefore be a salient barrier [49]. VILPA may therefore be a particularly suitable alternative for these individuals. Likewise, VILPA may be a good option for older adults, as aging stereotypes that are present in the fitness and gym settings [50] might prevent this population group from being physically activity in such settings.

Most themes were consistent across the three age groups, with the exceptions of perceptions of aging which was not noted in the young group, and gamification which was not identified in the oldest group. This homogeneity is somewhat surprising given the rather large age range (35–75 years). However, a systematic review examining barriers and motivation of physical activity also found that barriers were comparable amongst 50–64-year-old versus 65-70-year-old people, [7] suggesting that VILPA interventions targeting middle-aged and older adults may require limited differentiation.

### Implications and directions for future research

The results of our study demonstrate the potential utility and feasibility of promoting short, intense bouts of physical activity integrated into peoples’ existing everyday activities. The study demonstrated that VILPA appears to align better with the priorities, values and identities of physically inactive adults compared to other types of physical activity. Results pertaining to the barriers and enablers of VILPA facilitate identification of specific interventions strategies that could be used in VILPA-promoting interventions. Table 4 summarizes suggestions for how each barrier and enabler could be addressed in practice and research.

For example, perceptions of aging could be addressed by education focusing on challenging aging stereotypes [51]. The need for knowledge could be addressed by the development of specific guidelines on VILPA and ways in which to integrate VILPA into everyday activities. An improved sense of agency and self-efficacy in older adults may also be achieved by highlighting the importance of relative (increasing intensity over and above what they normally do), as opposed to absolute (comparing to others or their younger selves), intensity in the promotion of VILPA. The provision of environmental cues relevant to diverse settings could be important to include in interventions designed to increase VILPA. The use of prompts and reminders warrant intervention strategies that remind participants at the right time and place to engage in the behavior. Here, just-in-time-adaptive-interventions [52], which enables the delivery (e.g., via a mobile application) of targeted support at the “right time” when people need it the most, could be highly suitable. Further, gamification elements could be incorporated into mobile applications designed to increase VILPA. Finally, one way to develop routines is to use habit formation strategies that align with the desired behavior. Habits are formed when the behavior is performed repeatedly in the same context (e.g., time of day, or location) [53]. Thus, when people face the context, it becomes a cue that automatically triggers the behavior. Importantly, in the process of behaviors becoming habitual, the behavior is no longer dependent on goals and motivation but is triggered by context-response associations; thus, habits reduce the need to exert self-control. Some specific behavior change techniques can facilitate the formation of habits. One is the use of implementation intentions in which the person specifies where, when, and how to perform the behaviors [54], because it helps activate the automatic cue-response association. Another is the use of personally salient rewards because they can promote the repetition of the behavior which is an important component of habit formation [53]. These techniques could feasibly be incorporated into interventions designed to increase VILPA.

We previously argued that the identification of barriers and enablers of VILPA was an important first step to develop and design VILPA interventions. The next step is to design VILPA interventions that address barriers and leverage enablers identified in this study. Subsequently, the feasibility, acceptability and preliminary efficacy of these efforts should be assessed in feasibility, pilot, and efficacy trials, prior to being tested in definitive effectiveness trials. The feasibility trial should include explore issues associated with risk and safety of VILPA. Additionally, there is potential to develop and test the use of a smartphone application that incorporates the intervention strategies we have proposed based on our findings. Such a mobile application should be co-developed with the target population to ensure acceptability and undergo thorough testing regarding its efficacy as a tool to promote VILPA. Finally, recent developments in the measurement of habitual physical activity and VILPA via accelerometry and new algorithms [12, 55] open up new possibilities for research on VILPA. For example, the adherence effects of a VILPA intervention designed based on the results of the present study can be assessed. The detailed measurement of VILPA will also enable dose-response effects of VILPA interventions on health to be estimated. In addition, other contemporary methodological approaches could be considered to assess effects of VILPA, including ecological momentary assessment methodologies [56] and just-in-time-adaptive interventions [52].

### Strengths and Limitations

This is the first study to examine barriers and enablers of VILPA in physically inactive middle-aged and older adults. We specifically targeted and included only middle-aged and older adults who self-identified as physically inactive, which is critical given these are the populations in most need of increasing levels of physical activity [4, 5]. Their perspectives and experiences can inform interventions to increase VILPA. Further, the use of the COM-B framework enabled us to identify specific intervention targets which can be tested in future research.

Some limitations of our study should be considered in the interpretation of the findings. First, the use of the COM-B framework may have restricted our interpretation of the data to this framework. The use of alternative theories or frameworks might highlight other issues that could contribute to the understanding of barriers and enablers of VILPA. There was a higher proportion of females compared to males and the proportion of individuals who were non-white was small. A more diverse sample may have yielded different results. Finally, due to some drop-out among interviewees, the number of participants in each focus group was smaller than what is typically seen, potentially reducing the heterogeneity of discussion points among participants.

## Conclusions

Given the very low physical activity levels of middle-aged and older adults, novel approaches to gaining the health benefits of physical activity are urgently needed. VILPA, whose health potential is supported by recent epidemiological evidence, [12] may be a feasible option for people who are unable or unwilling to take part in structured exercise. Most of the barriers and enablers we generated in this study are theoretically modifiable. Promoting the time-efficient nature and simplicity of VILPA, requiring no equipment or special gym sessions, the use of prompts and reminders at opportune times, and habit formation strategies could capitalize on the enablers. Addressing the age-suitability and safety of the small bouts, the development of specific guidelines, addressing safety concerns, and explicating the potential benefits of, and opportunities for doing, VILPA could ameliorate some of the barriers identified. Finally, VILPA interventions targeting middle-aged and older adults may require limited customization, which speaks to the potential for such interventions to be delivered at scale.

## Electronic supplementary material

Below is the link to the electronic supplementary material.

Supplementary File 1: Physical Activity Screening Measure

Supplementary File 2: Interview Guide

Supplementary File 3: COREQ Checklist

## Data Availability

The datasets used and analyzed during the current study are available from the corresponding author on reasonable request.
