# Supplementary material for: Barriers and enablers of vigorous intermittent lifestyle physical activity (VILPA) in physically inactive adults: a focus group study
Source: Int J Behav Nutr Phys Act. 2023 Jul 4;20:78. doi: 10.1186/s12966-023-01480-8 (PMC10321001; doi:10.1186/s12966-023-01480-8)
Supplement: Supplementary file 1 — Physical Activity Screening Measure [file 12966_2023_1480_MOESM1_ESM.docx]

Additional File 1. Physical activity screening measure

1. In the past week, on how many days have you done a total of 10 minutes or more of physical activity, which was enough to raise your breathing rate? This may include sport, exercise, and brisk walking or cycling for recreation or to get to and from places, but should NOT include housework or physical activity that may be part of your job (1)

PLEASE ENTER NUMBER OF DAYS HERE _________

1. Do you currently participate in any regular exercise program (either on your own

or in a formal class) designed to improve or maintain your physical fitness?(2)

Yes  No

1. Do you suffer from any mental health illnesses that can make it difficult to complete an online questionnaires by yourself?

Yes  No

1. Is your current age between 35 and 75 years?

Yes  No

Instructions for researchers:

Eligible for participation if:

Q1 ≤ 3 AND Q2 = No Q3 = No Q4 =Yes

Q1 < 2 AND Q2 = Yes Q3 = No Q4 =Yes

Milton K, Bull FC, Bauman A. Reliability and validity of a single-item physical activity measure. Br J Sports Med. 2011;45:203-208.

1. Schechtman KB, Barzilai B, Rost K, et al. Measuring physical activity with a single question. Am J Public Health. 1991;81:771–3.
