# Supplementary material for: Barriers and enablers of vigorous intermittent lifestyle physical activity (VILPA) in physically inactive adults: a focus group study
Source: Int J Behav Nutr Phys Act. 2023 Jul 4;20:78. doi: 10.1186/s12966-023-01480-8 (PMC10321001; doi:10.1186/s12966-023-01480-8)
Supplement: Supplementary file 2 — Interview Guide [file 12966_2023_1480_MOESM2_ESM.docx]

**Supplementary File**

Mapping of interview topics to the COM-B domain

| **COM-B domain** | | **Interview topics** |
| --- | --- | --- |
| *Capability* | Physiological and psychological indicators of VILPA intensity: | |
|  | Experiences of doing VILPA | |
|  | What makes it difficult to do VILPA | |
| *Opportunity* | Situations in which participants perform VILPA | |
|  | Aspects of the environment (physical, social) that make it easier or more difficult to do VILPA | |
| *Motivation* | Types of VILPA that are liked versus disliked | |
|  | Reasons for doing VILPA | |
|  | Relevance of VILPA in daily life | |
|  | Feelings after doing VILPA | |
|  | Intentions to increase VILPA | |

**Specific interview questions**

1. What do you think about this concept of VILPA? How relevant are these activities in your daily life? [prompt: can you provide some examples?]
2. What does high intensity mean to you? How do you know if you have taken part in high intensity activities? [how does it feel: prompt physiological and behavioural signals]
3. Can you describe in some detail some activities that you would do as part of your daily life that you consider to be of high intensity? What would be the typical duration of these activities?
4. What do you see as the advantages for you to do more of these activities? What do you see as the disadvantages for you to do more of these activities?
5. What are some reasons why you would you do such high intensity activities? In which situations would you do them? How do they make you feel?
6. What types of high intensity activities do you like to do? Which ones don’t you like? Are there any that you try to avoid? Why?
7. What prevents you from doing more of these activities (use examples from earlier part of conversation)?
8. What would help you to do more of these activities/what would convince you to do more of these activities? [prompts: what people might say, support they could provide, could different aspects of the physical environment help, key opportunities during the day, places or situations that are conducive, cues and reminders]
